# Supplementary material for: Facile Synthesis of the Polyaniline@Waste Cellulosic Nanocomposite for the Efficient Decontamination of Copper(II) and Phenol from Wastewater
Source: Nanomaterials (Basel). 2023 Mar 11;13(6):1014. doi: 10.3390/nano13061014 (PMC10059074; doi:10.3390/nano13061014)
Supplement: Supplementary file 1 [file nanomaterials-13-01014-s001.zip › nanomaterials-2248215-supplementary.pdf]

nanomaterials

Facile Synthesis of the Polyaniline@Waste Cellulosic Nanocomposite for the Efficient Decontamination of Copper(II) and Phenol from Wastewater

Ahmed N. Doyo, Rajeev Kumar * and Mohamed A. Barakat *

Department of Environmental Sciences, Faculty of Meteorology, Environment and Arid Land Agriculture, King Abdulaziz University, Jeddah 21589, Saudi Arabia
* Correspondence: rsingh@kau.edu.sa (R.K.); mababdullah1@kau.edu.sa (M.A.B.)

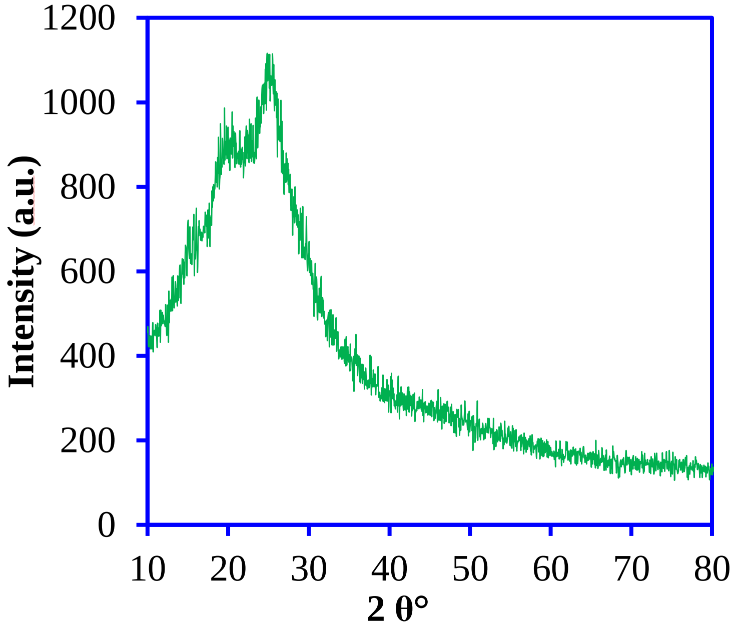

Figure S1. XRD pattern of the polyaniline.

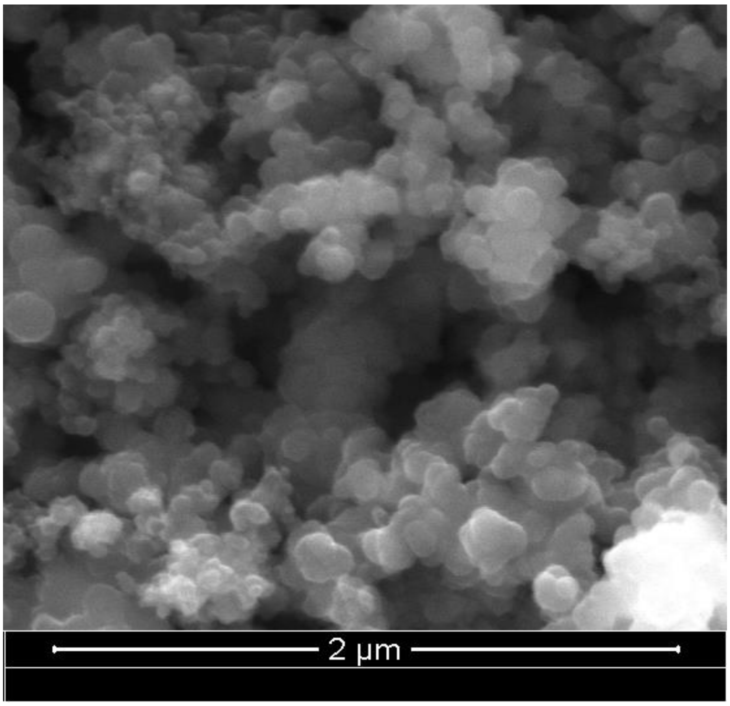

Figure S2. SEM image of the polyaniline.

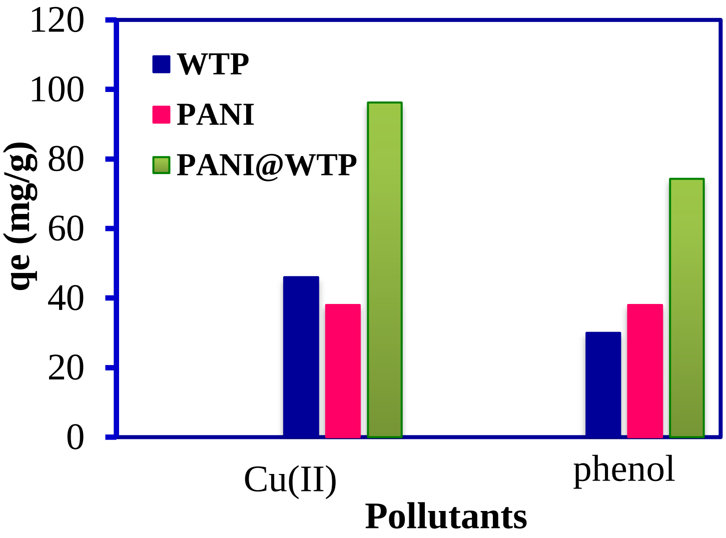

Figure S3. The comparison of adsorption capacities of WPT, PANI and PANI@WTP for Cu(II) and phenol removal. (concentration; 100 mg/L, adsorbent dosage; 0.02g, volume; 20 mL, pH 5.2 for Cu(II), pH, 5 for phenol, Temp.; 30 °C).
